# Cost Reductive Laparoendoscopic Single Site Surgery Endotrainer and Animal Lab Training—Our Methodology

**DOI:** 10.1155/2010/598165

**Published:** 2010-02-18

**Authors:** Manickam Ramalingam, Kallappan Senthil, Anandan Murugesan, M. G. Pai

**Affiliations:** ^1^Department of Urology, PSG Institute of Medical Sciences & Research, Peelamedu, Coimbatore 641004, India; ^2^Urology Clinic, 3 Gowtham Annexe, 1054 Avinashi Road, Coimbatore 641 018, India

## Abstract

Laparoendoscopic single site surgery (LESS) is a new avenue in laparoscopic urology. The main advantage is the enhanced cosmetic benefits of single hidden scar. Lately many papers are being published on various procedures done by LESS. Like conventional laparoscopy, this approach is likely to be used more widely and hence exposure to this field is essential. However, formal training in this technique is not widely available. Expensive ports and nonavailability of endotrainer may be the factors deterring the training. We have modified the standard laparoscopic endotrainer with improvised ports, to make it suitable for single port laparoscopic training. For the animal lab training improvised ports and low cost instruments were used. Thus the overall cost of the training in LESS was reduced, and better confidence levels were achieved prior to human applications.

## 1. Introduction

Laparoendoscopic single site surgery (LESS) is a new avenue in laparoscopic urology. The main advantage is the enhanced cosmetic benefits of single hidden scar [1]. Lately many papers are being published on various procedures done by LESS [2]. Like conventional laparoscopy, this approach is likely to be used widely in future and hence exposure to this field is essential. However, formal training in this technique is not available. Expensive ports and nonavailability of endotrainer may be the factors deterring the training. We have modified the standard laparoscopic endotrainer. Port was improvised, to make it suitable for this procedure. For the animal lab training improvised ports and low cost instruments were used. Thus the overall cost of the training in LESS was reduced and better confidence levels were achieved prior to human applications.

## 2. Materials and Methods

**Figure 1:**
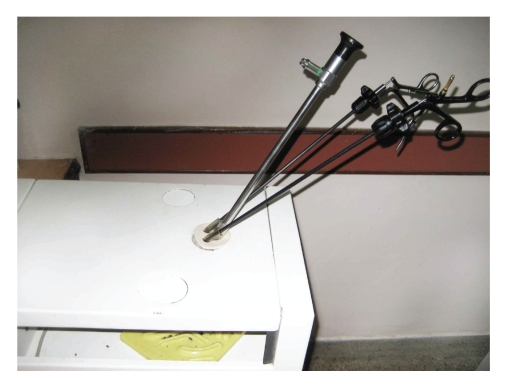
LESS endotrainer with modified port (1).

**Figure 2:**
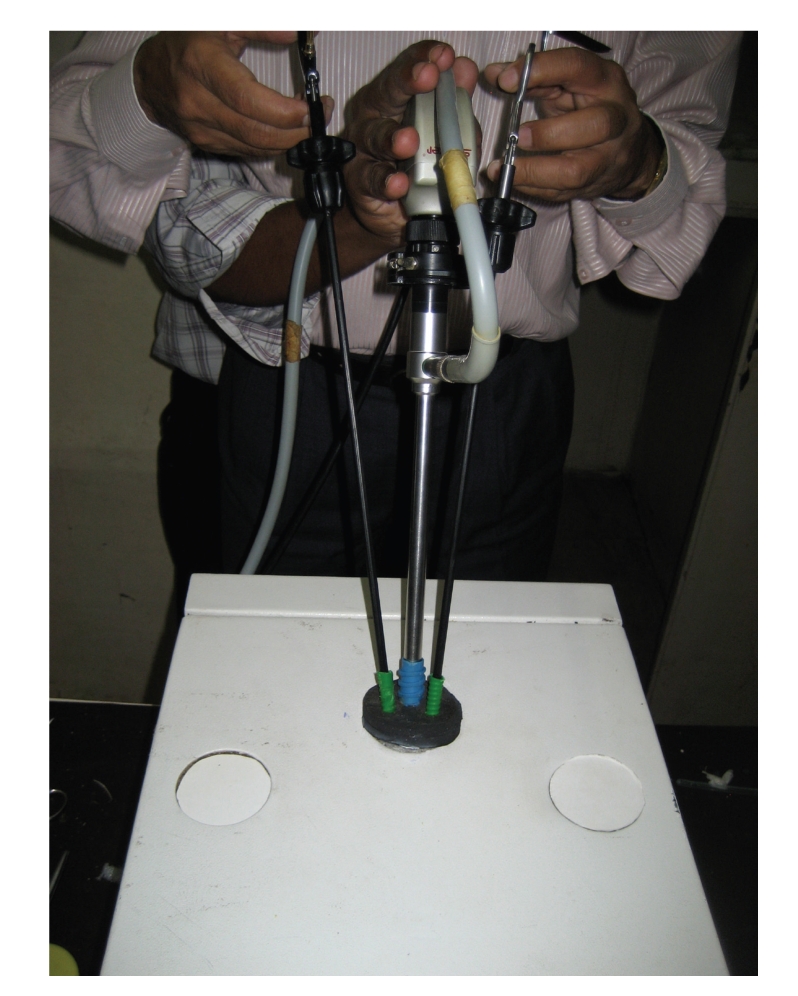
Modified LESS endotrainer port (2) being used for training.

**Figure 3:**
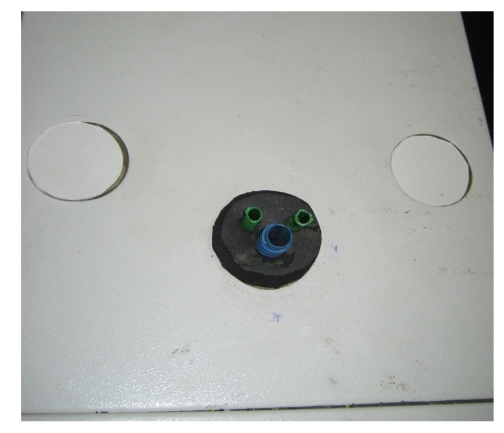
Modified LESS endotrainer port (2).

**Figure 4:**
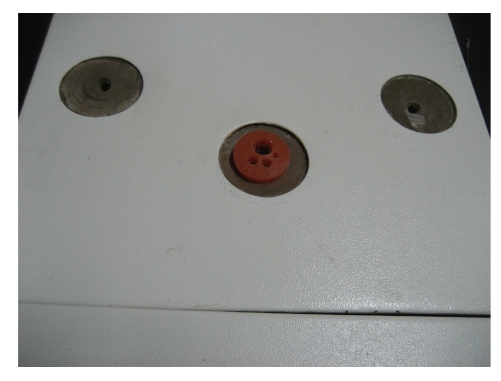
Endotrainer in our center with modified single port (3).

**Figure 5:**
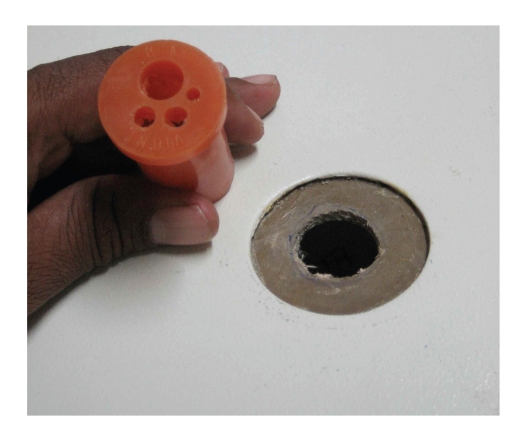
Standard endotrainer with modified port (3).

**Figure 6:**
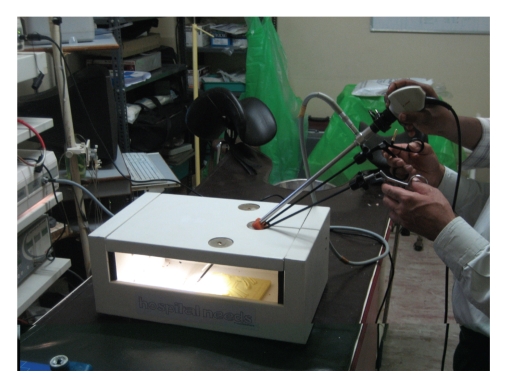
LESS Endotrainer with modified port (3). Note the crowding of instruments.

**Figure 7:**
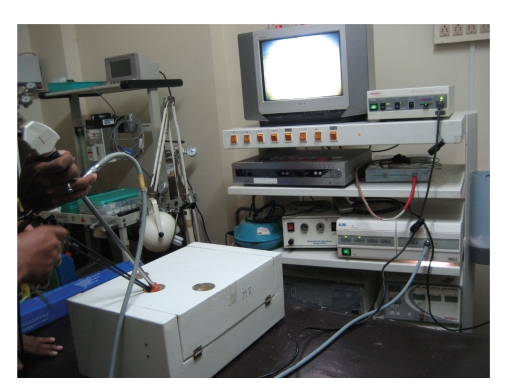
LESS Endotrainer setup.

**Figure 8:**
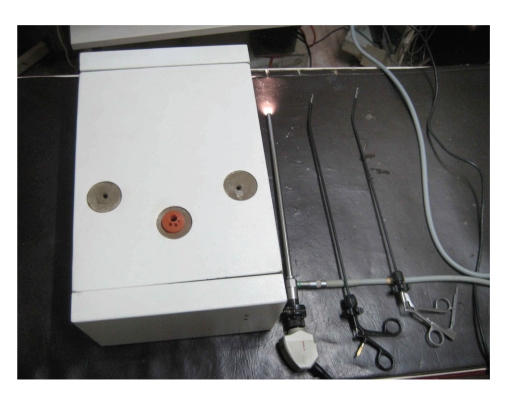
Single port endotrainer with angulated instruments.

**Figure 9:**
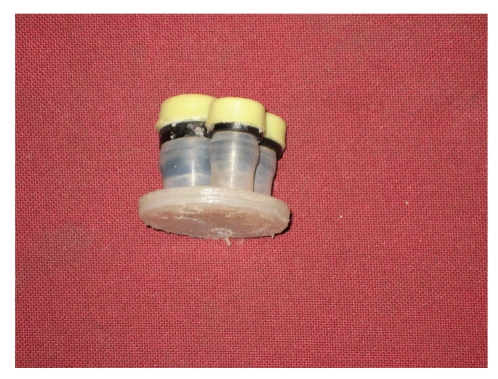
Modified LESS port (4) lateral view.

**Figure 10:**
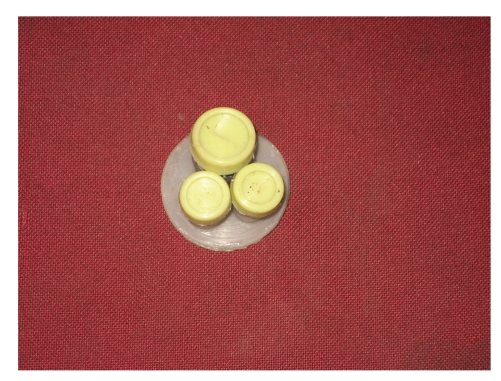
Modified LESS port (4).

*The LESS Endotrainer*. The endotrainer we have designed is a cuboidal box with the dimensions of 35 cm × 28 cm × 18 cm. These dimensions are developed, such that, they nearly correspond to the normal adult peritoneal cavity. The space is adequate for the placement of instruments and training objects. The standard laparoscopic endotrainer consists of three port sites. For single port training, we modified the central port with a multiport system measuring 2.5 cm in diameter. Various materials like rubber sheet for the multiport platform, and plastic tubes or short used 10 mm and 5 mm plastic ports for the trocars were used. Once assembled, this multiport system consisted of a 10 mm port for insertion of endocamera and two 5 mm ports for insertion of hand instruments (Figures 1, 2, 3, 4, 5, 6, 7, 8, 9 and 10). The endocamera could be replaced by a webcamera as in the standard endotrainer box when there is no assistant.

## 3. Instruments and Training

The instruments for LESS training are designed with an angulation in the distal end to prevent the clashing of instruments. Angulated Maryland dissector and curved scissors without angulation are used for dissection. Standard needle holder is used for suturing. The reticulating instruments are also used during the training.

**Figure 11:**
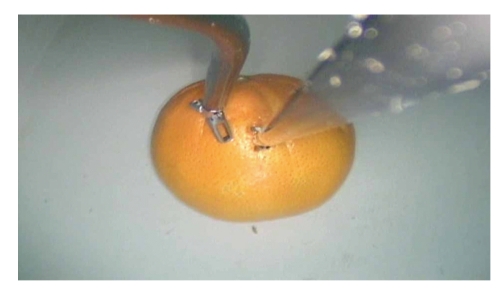
Orange dissection.

**Figure 12:**
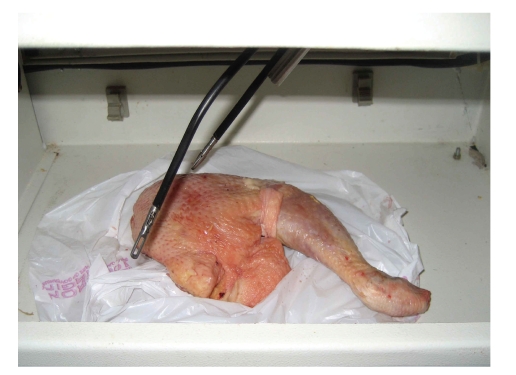
Chicken dissection in the LESS endotrainer.

**Figure 13:**
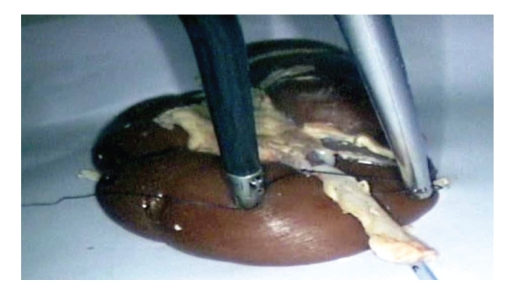
Suturing in Bovine kidney.

Training pattern for single port laparoscopy was similar to conventional laparoscopic training. Hand eye coordination, dissection, and suturing exercises were carried out. The hand eye co-ordination exercises included transfer of objects between the instruments and transfer between bowls placed within the trainer. The technique of dissection was by peeling orange skin (Figure 11) and dissecting a chicken leg model (Figure 12). Suturing was practiced on chicken leg and cadaver bovine kidney ureteropelvic junction (UPJ) (Figure 13). 

**Figure 14:**
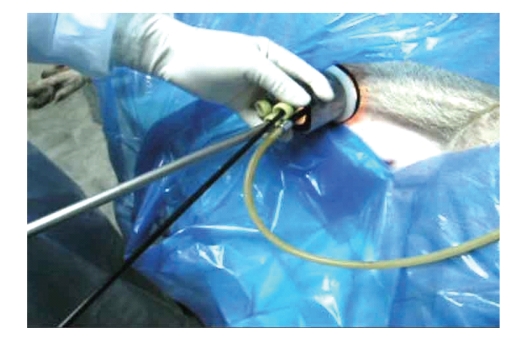
LESS in live pig—in progress.

**Figure 15:**
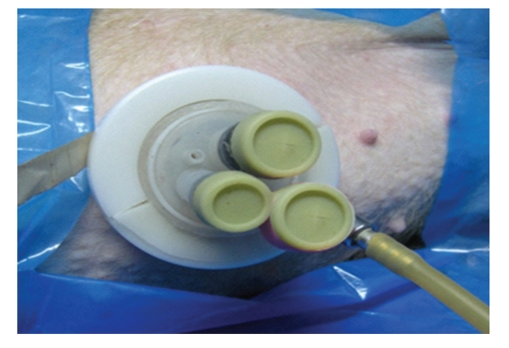
Modified single port in live animal.

*Evaluation of training*. We conducted training sessions among 3 of our faculty, who are experienced laparoscopic surgeons, using our LESS endotrainer. A total of 10 sessions were carried out by each of the consultants over a period of 10 days. Objective assessment was done using the task completion time (TCT) [3] as the unit of measurement. Complete peeling of orange and suturing of UPJ in cadaver bovine kidney (six interrupted sutures) were the two tasks assessed at the first, sixth, and tenth sessions. After completion of the endotrainer sessions, animal lab training was carried out on a live anesthetised pig using single port (Figures 14 and 15).

## 4. Results

**Figure 16:**
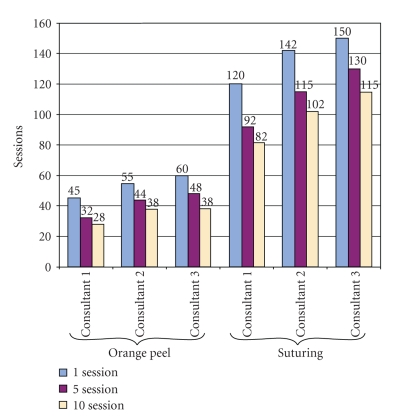
Task Completion Time—Chart.

The results are summarized in the form of a chart (Figure 16). The results showed that with each passing session the subjective difficulty of the procedure decreased. Statistical analysis was done using SPSS software. The average TCT decreased as the sessions progressed from the first attempt to fifth attempt and tenth attempt. The correlation coefficient of the TCT was -0.8 and -0.9 for orange peel and bovine model suturing, respectively. This shows that, with the progression of sessions, the task completion time significantly decreases. The difference in the TCT of both orange peeling and suturing between sessions 1, 5, and 10 was highly significant (*P* < .0001) (Table 1). 

After 10 endotrainer sessions pig nephrectomy was attempted. The average duration was around 90 minutes. The average duration of conventional laparoscopic nephrectomy in the porcine model in the past used to be about 45 minutes as per the previous records of our faculty. The major difficulty encountered by the surgeons was the clashing of instruments and their orientation. No major complications were encountered during the procedure. Hemostasis was ensured using monopolar electrocautery.

## 5. Discussion

Wickham introduced the field of Laparoscopy to Urology [4] by performing the first laparoscopic ureterolithotomy in 1979. However laparoscopy gained momentum only after Clayman did a laparoscopic simple nephrectomy in 1991 [5]. Today more and more ablative and reconstructive laparoscopic procedures are performed widely. With experience and continuous training, the duration of surgeries got significantly reduced [6]. With the increasing interest on cosmesis, the sizes of the ports and instruments were reduced from 12 mm to 1.8 mm [7]. To make this further cosmetically appealing and minimally invasive, new avenues like natural orifice transluminal endoscopic and single site laparoscopic surgery (LESS) evolved. 

LESS was introduced in Urology by Gettman et al. in 2002 by performing a transvaginal laparoscopic nephrectomy in a porcine model [8]. The first paper on LESS in humans was published by Raman et al. in 2007 [9]. Since then few centers are performing more and more of LESS procedures. In the present scenario, the duration of surgery for LESS is considerably prolonged compared to conventional laparoscopy [10]. 

With adequate training and experience, LESS can also be performed more widely. However compared to conventional laparoscopic training programmes, organized training programmes for LESS is not available widely at the moment. Endotrainers are also freely available for the conventional laparoscopic training. However, to our knowledge, no literature has been published on the use of endotrainers for LESS. This may be because of the cost of the single ports resources, and mentor availability. Further more, animal lab training without dry lab may not be ethically acceptable and can be too tiring.

Hence we have modified the conventional multiport endotrainer to a single port endotrainer for LESS training in our training programme. The cost of conversion was very minimal. The multiport and instruments for training in LESS are improvised from locally available resources. With an extra cost of 50$, the standard endotrainer could be modified to a single port trainer.

From the training among our faculty, we could appreciate that LESS was more difficult than conventional laparoscopy, even after multiple training sessions. The operating durations for the dissection and suturing both are significantly higher for LESS. We have not compared LESS training with conventional laparoscopic training in terms of trainee surgeons versus experienced surgeons.

The main disadvantage of LESS was clashing of instruments because of the closely placed ports. As triangulation of instruments is not possible; suturing needs extensive training. The transmission of the pressure and tactile feedback during dissection is different. Occasionally the right-handed angulated instrument may be on the left side of the left-handed instrument and vice versa necessitating proper orientation of instruments (crossing within the endotrainer and within abdominal cavity). These were detrimental factors in learning LESS.

All our faculties could complete the nephrectomy using single port in live animal lab with a prolonged operating time.

## 6. Conclusion

LESS training in endotrainer is preferable prior to training in animal lab and subsequent human surgery. Multiple sessions of LESS endotrainer practice will help to overcome the difficulties and reduce the operating time in LESS procedures. This specially designed endotrainer is a cost reductive step in LESS training programme.

## Figures and Tables

**Table 1:** Task completion time—Results.

Task	Session	Mean duration	Variance	SD	*P*-values
Orange peel	1	53.33	58.33	7.6	
	5	41.33	69.33	8.3	.0009
	10	34.66	33.33	5.73	

Suturing	1	137.33	241.33	15.5	
	5	112.33	366.33	19.1	.0005
	10	99.66	276.33	16.6	
